# Is the Efficiency of RNA Silencing Evolutionarily Regulated?

**DOI:** 10.3390/ijms17050719

**Published:** 2016-05-12

**Authors:** Kumiko Ui-Tei

**Affiliations:** 1 Department of Biological Sciences, Graduate School of Science, The University of Tokyo, 7-3-1 Hongo, Bunkyo-ku, Tokyo 113-0033, Japan; ktei@bs.s.u-tokyo.ac.jp; Tel.: +81-3-5841-3043; 2 Department of Computational Biology and Medical Sciences, Graduate School of Frontier Sciences, The University of Tokyo, 5-1-5 Kashiwanoha, Kashiwa-shi, Chiba-ken 277-8561, Japan

**Keywords:** RNA silencing, siRNA, miRNA, silencing efficiency, base-pairing stability, thermodynamic stability

## Abstract

Small interfering RNAs (siRNAs) and microRNAs (miRNAs) regulate gene expression in a sequence-specific manner. Genes with partial complementarity to siRNA/miRNA sequences in their 3′-untranslated regions (UTRs) are suppressed by a mechanism referred to as the siRNA off-target effect or miRNA-mediated RNA silencing. However, the determinants of such RNA silencing efficiency are poorly understood. Previously, I and co-workers reported that the efficiency of RNA silencing is strongly correlated with the thermodynamic stability of base pairing in the duplex formed within an siRNA/miRNA and between the seed region and its target mRNA. In this review, I first summarize our previous studies that identified the thermodynamic parameter to estimate the silencing efficiency using the calculated base pairing stability: siRNAs downregulate the expression of off-target genes depending on the stability of binding between the siRNA seed region (nucleotides 2–8) and off-target mRNAs, and miRNAs downregulate target mRNA expression depending on the stability of the duplex formed between the 5′ terminus of the miRNA and its target mRNA. I further discuss the possibility that such thermodynamic features of silencing efficiency may have arisen during evolution with increasing body temperature in various organisms.

## 1. Introduction

MicroRNAs (miRNAs) are a large family of small non-coding RNAs (ncRNAs) that repress gene expression by inhibiting translation and promoting mRNA decay. More than 2000 miRNAs have been identified in human cells; they are predicted to regulate the activity of hundreds of protein-coding genes to control various aspects of biological processes, including development, differentiation, proliferation, antiviral defense, and metabolism. They also act as tumor suppressors or oncogenes and are often dysregulated in tumors. The target genes of miRNAs can be computationally predicted based on experimentally validated algorithms [1,2,3]. However, to understand the regulation of gene expression mediated by miRNA silencing, it is essential to predict the silencing efficiency of each miRNA accurately in addition to its target genes.

**Figure 1:**
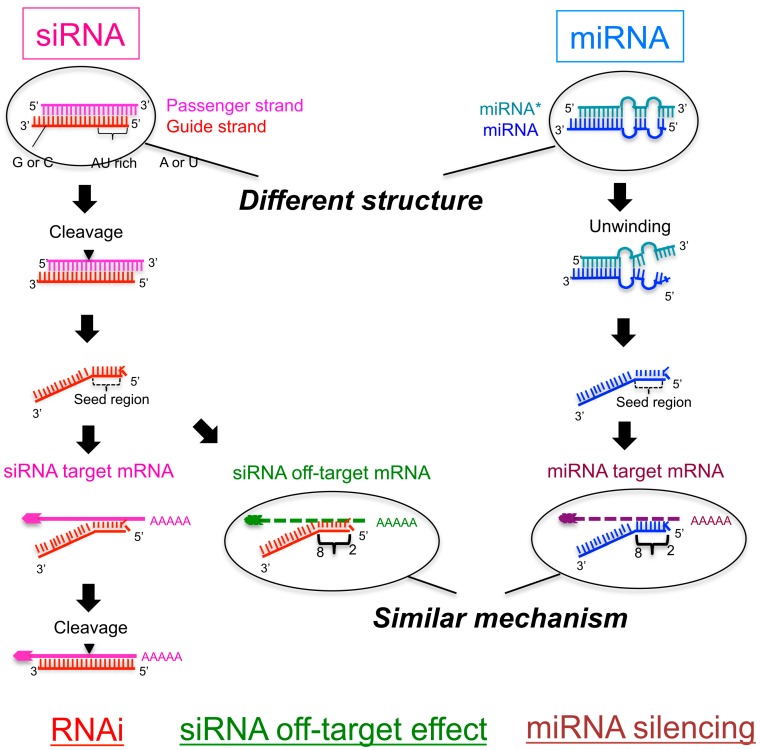
Schematic figure showing the pathway of small interfering RNA (siRNA)-mediated RNA interference (RNAi) and microRNA (miRNA)-mediated RNA silencing. The mechanisms of downregulation of off-target genes in the RNAi pathway and that of target genes in miRNA-mediated RNA silencing are similar: The seed region of the siRNA/mRNA recognizes the 3′-UTRs of off-target or target genes and downregulates their expression. The efficiency of the off-target effect by functional siRNAs is dependent on the thermodynamic stability of binding between the seed region and the off-target transcript. However, miRNA-mediated silencing efficiency is dependent on the thermodynamic stability of binding in both the seed-target duplex and 5′-terminal miRNA duplex, probably because the secondary structure of the miRNA is different from that of the siRNA.

Primary miRNAs (pri-miRNAs) are sequentially processed by the double-stranded RNA cleavage enzymes Drosha and Dicer. Drosha cleaves pri-miRNAs to generate precursor-miRNAs (pre-miRNAs) in the nucleus [4,5,6]; these pre-miRNAs are further processed by Dicer to generate ~22-nucleotide (nt) miRNA duplexes in the cytoplasm [5,7,8]. The miRNA duplex is loaded onto Argonaute (AGO), creating an RNA-induced silencing complex (RISC) [9,10,11,12,13], and subsequently unwound into a single-stranded mature miRNA in the RISC [12,13]. The retained RNA strand acts as a guide to recruit the RISC to the 3′-untranslated region (UTR) of a target mRNA with complementarity to the seed region, at positions 2–8, to promote translational repression [14,15,16] (Figure 1).

Another type of small ncRNA, small interfering RNA (siRNA), is widely used as a tool of loss-of-function experiments. The siRNA is usually introduced into cells exogenously to reduce the expression of genes as double-stranded RNAs ~21 nt in length with 2-nt 3′ overhangs. The guide strand of the siRNA shares full sequence complementarity with the coding sequence of its intended target mRNA and triggers enzymatic cleavage of the mRNA by AGO, the catalytic component of the RISC, between nts 10 and 11 via RNA interference (RNAi) [17,18,19,20,21,22] (Figure 1). In addition, the siRNA guide strand recognizes many mRNA 3’-UTRs with only partial complementarity to the seed region and downregulates their expression. This effect is referred to as an off-target effect (Figure 1). Such off-target effects are considered to be induced through a mechanism similar to that of target silencing by miRNA [2,3,23]. Thus, miRNA-mediated silencing and siRNA-based off-target effects are thought to share similar silencing machinery through common target recognition processes using the seed region.

## 2. The Silencing Efficiency of siRNAs on Off-Target mRNAs Is Regulated by the Stability of Base Pairing between the siRNA Seed Region and Off-Target mRNA

**Figure 2:**
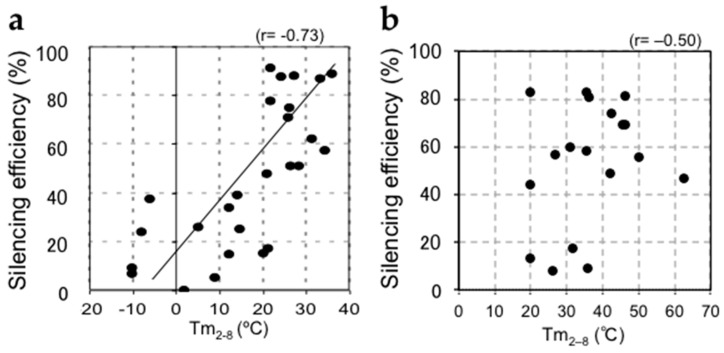

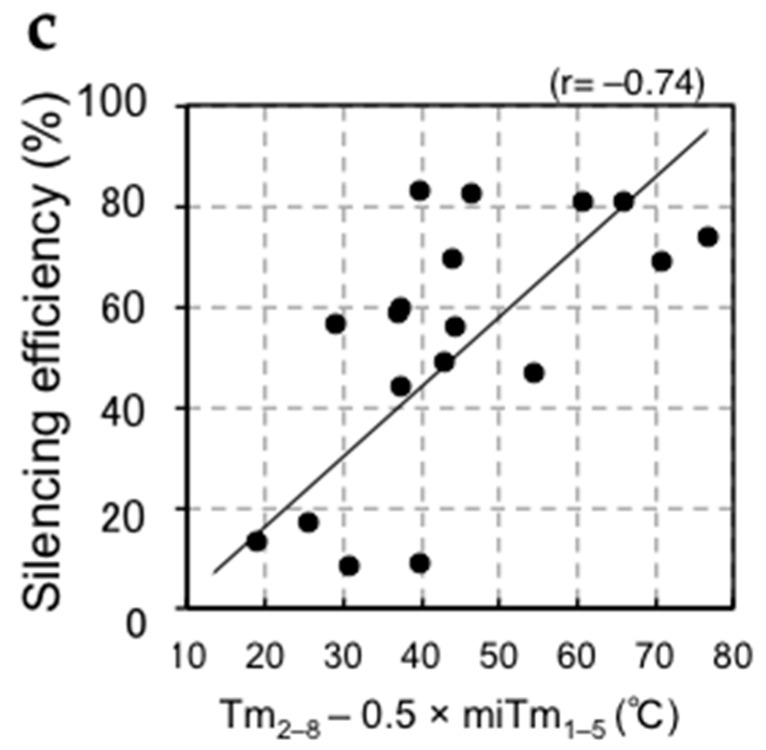
Relationships between silencing efficiency and thermodynamic parameters. The *y*-axis means the silencing efficiency, indicating the value (%) subtracted the measured luciferase activity (%) from 100. (**a**) The calculated *T*m-value of the duplex formed between the siRNA seed region and target mRNA (*T*m_2–8_) showed a positive correlation with siRNA-mediated silencing efficiency; (**b**) The calculated *T*m_2–8_ values showed a marginal correlation with miRNA-mediated silencing efficiency; (**c**) The miRNA-mediated silencing efficiency showed a strong positive correlation with the parameter composed of *T*m_2–8_ and miRNA 5′-terminal stability (mi*T*m_1–5_), shown as *T*m_2–8_ − 0.5 × mi*T*m_1–5_.

The mechanism of siRNA-induced off-target effects is similar to miRNA-mediated target RNA silencing, which regulates the expression of many unintended transcripts with partial complementarity. The 3′-UTRs of the off-target transcripts are complementary to the guide strand seed region. In the RISC, the seed nucleotides of the guide strand loaded onto AGO are present on its surface in a quasi-helical form to serve as the entry or nucleation site for off-target mRNAs [24,25]. I and co-workers clarified the molecular basis determining the efficiency of seed-dependent off-target silencing in terms of the stability of the protein-free RNA duplex [26] (Figure 1). In a previous study, we used siRNAs with A or U residues at position 1 from the 5′ end of the siRNA guide strand, three to six A/U residues in nt positions 2–7, and a G/C at position 19, as these siRNAs are highly functional [27]. The guide strand of such highly functional siRNA is expected to be incorporated into the RISC very effectively by unwinding into single-stranded RNA from its 5′ terminal. Similarly, other groups also reported that the functional siRNAs have such asymmetry in base pairing stability of the two terminals [28,29]. Thus, such asymmetry may be one of the essential features for the functional siRNAs. However, the terminal asymmetry did not control the “efficiency” of the siRNA off-target effect. Therefore, to find out the determinant of the “efficiency” of the siRNA off-target effect, we used siRNAs satisfying all of the highly functional siRNA characteristics identified in our previous report [27]. Using these siRNAs, we measured the siRNA off-target effects by a luciferase reporter assay and microarray experiments. In the luciferase reporter assay, each seed complementary sequence was inserted into the 3′-UTR region of the luciferase gene, and the luciferase activity, the indicative of the expression level, was measured with a luminometer after the transfection of each siRNA. The results revealed that the seed-dependent off-target efficiency was positively and negatively correlated with the melting temperature (*T*m) (*r* = 0.74, Figure 2a) and standard free energy change (Δ*G*) (*r* = −0.69), respectively, calculated by the nearest-neighbor procedure [26] for the formation of the duplex between the siRNA seed region (positions 2–8) and the complementary 3’-UTR sequence in the off-target transcript [27]. Thus, our results indicated that the high base pairing stability shown as *T*m_2–8_ in the duplex formed between the siRNA seed region and the target 3’-UTR strongly reduced off-target transcript expression, but that the low stability exerted a very weak effect.

The main factor regulating off-target silencing efficiency is considered to be the thermodynamic stability of base pairing between the siRNA seed region and off-target transcript. However, considerable deviation was observed for the off-target efficiencies measured by the reporter assay even when the same siRNA was used. These results suggested that the non-seed region sequence may also slightly affect silencing efficiency. We recently demonstrated that there are additional auxiliary factors that regulate the degree of downregulation of off-target transcripts [30]; both the *T*m-value of the siRNA non-seed region at positions 8–15 and the GC contents of its corresponding off-target transcripts are negatively correlated with the off-target silencing efficiency.

## 3. The Efficiency of Silencing of Target mRNAs by miRNAs Is Regulated by the Stability of Base Pairing in the 5′ Region of the miRNA and that between the miRNA Seed Region and Target mRNAs

Target mRNAs are recognized by miRNAs via a mechanism similar to the siRNA off-target effect, and target mRNAs complementary to the miRNA seed region are mainly downregulated [2,3,23]. However, unlike siRNA off-target silencing, Hibio *et al.* previously showed that the efficiency of miRNA-mediated silencing was not simply correlated with the *T*m-value of the seed-target duplex (Figure 2b) [31]. The most prominent differences between siRNAs and most miRNAs are the structural features of the duplex—siRNAs usually form completely complementary duplexes, while most miRNA duplexes have internal bulges or mismatches. Hibio *et al.* [31] evaluated the involvement of miRNA duplexes formed in any given region by quantifying the base pairing stability by calculating *T*m-values incorporating the parameters of internal bulges and/or mismatches assigned as the *T*m-value in the miRNA duplex (mi*T*m). Luciferase reporter assays were performed to determine the optimal miRNA duplex region for the silencing efficiency. Our results indicated that the silencing efficiency of an miRNA is principally determined by the combinatorial thermodynamic parameters of *T*m_2–8_ (*T*m-value between the miRNA seed region at positions 2–8 and its perfectly complementary target sequence) and mi*T*m_1–5_ (*T*m-value of the miRNA duplex at positions 1–5), and that miRNA-mediated silencing efficiency is strongly correlated with the following formula: *T*m_2–8_ − 0.5 × mi*T*m_1–5_ (*r* = 0.74, Figure 2c) [31]. In this formula, *T*m_2–8_ reflects the base pairing stability in the seed-target duplex; strong (weak) base pairing stability may result in strong (weak) silencing efficiency. Positions 1–5 correspond to the 5′-terminal end of the miRNA, which is essential to determine the direction of miRNA unwinding. An miRNA molecule with low stability can be easily unwound into single-stranded RNA from the 5′ terminus and incorporated into the RISC [27,28,29]. Thus, miRNAs with low mi*T*m_1–5_ values may enhance target RNA silencing according to the ease of unwinding, while those with a high mi*T*m_1–5_ may repress target RNA silencing according to the difficulty of unwinding. Thus, although positions 2–5 overlap between the seed region and 5′-terminal region (1–5), the results of Hibio *et al.* indicated that the mi*T*m_1–5_ and *T*m_2–8_ values have opposite effects. In addition, it was necessary to subtract the value of mi*T*m_1–5_ from that of *T*m_2–8_ to measure the silencing efficiency; a high *T*m_2−8_ value and low mi*T*m_1–5_ value will increase the silencing activity, while low *T*m_2–8_ and high mi*T*m_1–5_ values decrease the silencing activity. Furthermore, the factor 0.5 for mi*T*m_1–5_ indicates that the effect of *T*m_2–8_ on silencing efficiency is about two-fold stronger than that of mi*T*m_1–5_.

**Figure 3:**
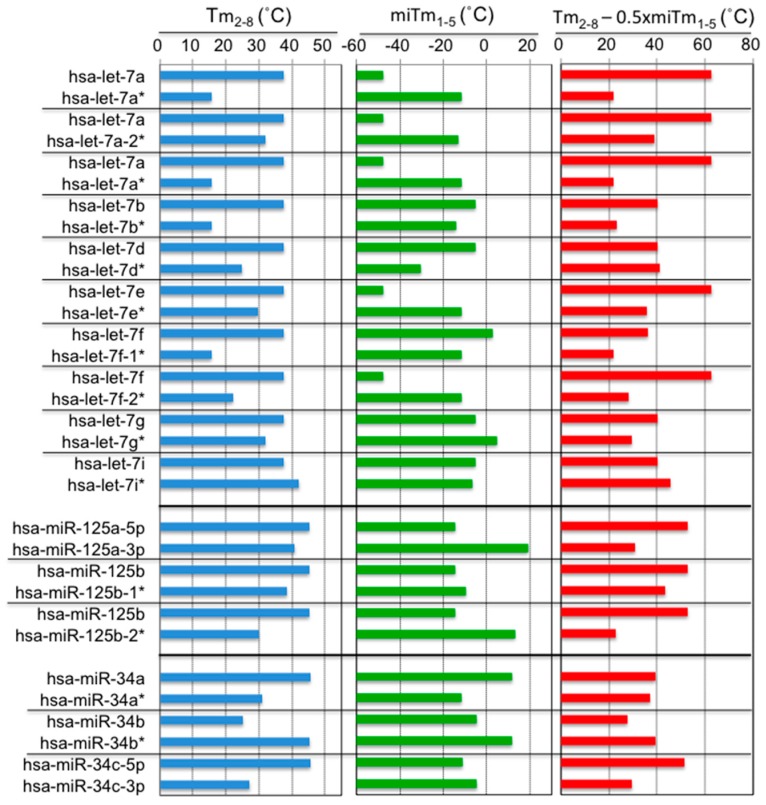
Calculated *T*m_2–8_, mi*T*m_1–5_, and *T*m_2–8_ − 0.5 × mi*T*m_1–5_ values for the miRNA families let-7, miR-125, and miR-34.

In miRBase (Available online: http://www.mirbase.org/), two miRNAs originating from the same predicted precursor are often annotated. Among them, the predominantly expressed miRNA is referred to as miR-xxx, and the opposite arm of the precursor is designated as miR-xxx*, as shown in Figure 3. Hibio *et al.* [31] calculated the *T*m_2–8_ and mi*T*m_1–5_ values for each side of the miRNA from 16 sets of miRNA duplexes (Figure 3). Interestingly, most of the predominant miRNAs showed higher *T*m_2–8_ values (14/16, 88%) and lower mi*T*m_1–5_ values (11/16, 69%) compared to the opposite miRNAs, and the resultant *T*m_2–8_ − 0.5 × mi*T*m_1–5_ values increased more (13/16, 81%) than the opposite miRNAs. Thus, although positions 2–5 overlapped between seed positions 2–8 and the 5′ terminus (positions 1–5), the predominant miRNA may form a secondary structure to reduce the 5′ terminal *T*m-value in the miRNA duplex, even if the *T*m-value of the perfect complementary duplex formed between its seed region and the target mRNA is high. Thus, miRNAs may regulate silencing efficiency by changing the secondary structure of their own duplex to reduce the base pairing stability, especially in the 5′-terminal region.

In addition to positions 1–5, a weak but significant correlation between silencing efficiency and the thermodynamic profile of the miRNA duplex was also observed at positions 13–17 [30]. Thus, weak stability at non-seed positions 8–15 may increase the miRNA-mediated silencing efficiency.

## 4. The Base Pairing Stabilities of miRNAs in Various Species are Strongly Correlated with Body Temperature

RNA silencing mediated by miRNAs is a conserved phenomenon in a broad range of metazoans [32]. We found that the silencing efficiency of miRNAs could be determined by the thermodynamic properties of protein-free RNA duplexes, *T*m_2–8_ and mi*T*m_1–5_. As the state of nucleotide base pairing is regulated by thermodynamic properties, growth temperature is important to regulate the silencing efficiency of miRNAs. An RNA duplex with low thermodynamic stability is not formed in the cells of an organism growing at a high temperature, but such a duplex could be formed at a low growth temperature. In contrast, an RNA duplex with high thermodynamic stability would not be unwound into single-stranded RNAs at low temperatures, although it could be unwound at high temperatures. Therefore, it is likely that organisms growing at high temperatures will have miRNAs with strong base pairing stability, while those with low growth temperatures will have miRNAs with weaker base pairing.

**Figure 4:**
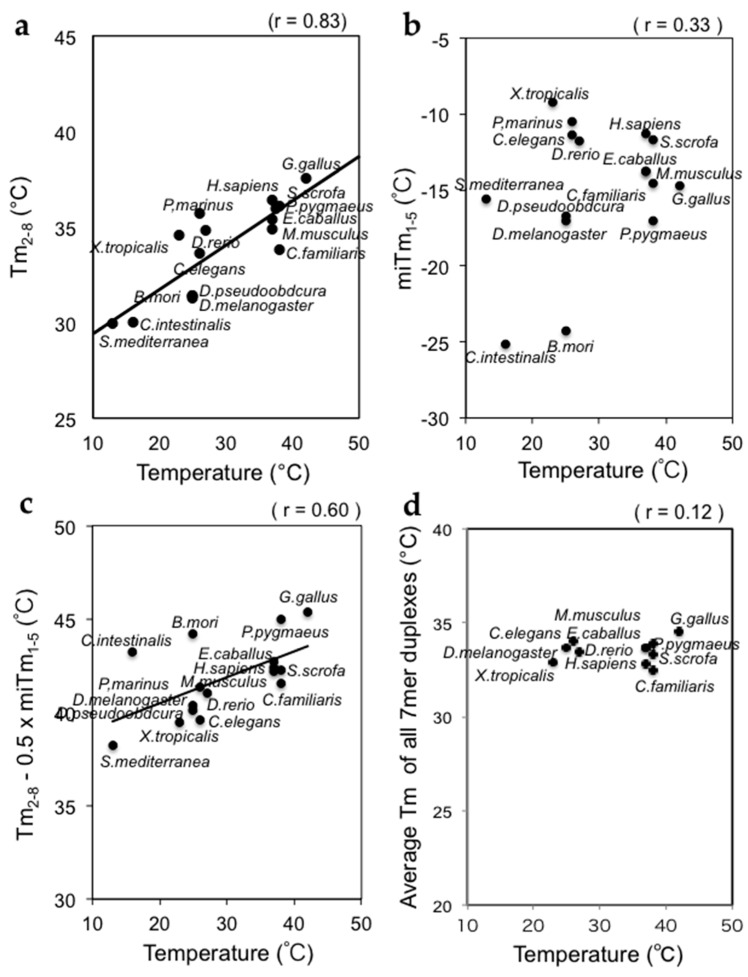
Strong correlations between the silencing parameters *T*m_2–8_ and mi*T*m_1–5_, and the growth temperatures of various organisms. (**a**) A strong correlation was found between *T*m_2–8_ and growth temperature; (**b**) A marginal correlation was found between mi*T*m_1–5_ and growth temperature; (**c**) A strong correlation was found between *T*m_2–8_ − 0.5 × mi*T*m_1–5_ and growth temperature; (**d**) Almost no correlation was found between the average *T*m-values for all 7-mer tRNA sequences and growth temperature.

We calculated the thermodynamic parameters of miRNAs from 16 different organisms registered in miRBase: *T*m_2–8_, mi*T*m_1–5_, and *T*m_2–8_ − 0.5 × mi*T*m_1–5_. The average *T*m_2–8_ values varied from 30 to 38 °C in these organisms (Figure 4a). The planarian *Schmidtea mediterranea* and ascidian *Ciona intestinalis* are heterothermic animals, but they are usually maintained at temperatures of approximately 10–15 °C. The African frog *Xenopus tropicalis*, the nematode *Caenorhabditis elegans*, the silkworm *Bombyx mori*, *Drosophila melanogaster*, *Drosophila pseudoobscura*, the lamprey *Petromyzon marinus*, and the zebrafish *Danio rerio* are reared at 23–27 °C. Homothermic animals such as humans (*Homo sapiens*), mice (*Mus musculus*), dogs (*Canis familiaris*), horses (*Equus caballus*), orangutans (*Pongo pygmaeus*), and pigs (*Sus scrofa*) have body temperatures of about 37 °C. The body temperature of chicken (*Gallus gallus*) is the highest at about 42 °C. Our results clearly showed that the average *T*m_2–8_ values of animals grown at a low temperature were low, while those grown at higher temperatures were high (Figure 4a). The correlation coefficient was high at *r* = 0.83.

The average mi*T*m_1–5_ values for the miRNAs of 16 different organisms were also calculated. The values varied from −25 to −9 °C (Figure 4b). With the exception of *B. mori* (mi*T*m_1–5_ = −23.0 °C) and *C. intestinalis* (mi*T*m_1–5_ = −22.2 °C), which showed significantly low values, the mi*T*m_1–5_ values of the organisms were low and similar (around −15 to −10 °C). The correlation coefficient between the mi*T*m_1–5_ values and growth temperatures was very low at *r* = 0.44 (Figure 4b).

The thermodynamic parameters of miRNA silencing efficiency, *T*m_2–8_ – 0.5 × mi*T*m_1–5_ values, were calculated using miRNAs from 16 organisms. The values varied less from 38 to 44 °C (Figure 4c). Although the values for *B. mori* (*T*m_2–8_ – 0.5 × mi*T*m_1–5_ = 44.2 °C) and *C. intestinalis* (*T*m_2–8_ − 0.5 × mi*T*m_1–5_ = 43.2 °C) were high compared to those organisms with similar body temperatures, other organisms showed strong correlations, with a correlation coefficient for the 16 organisms of *r* = 0.60 (Figure 4c).

As a control, the average *T*m-values of all 7-mer duplexes in tRNA sequences were calculated, and almost no correlation was observed (*r* = 0.12, Figure 4d).

These observations clearly supported the idea that the organisms with high growth temperature have miRNAs with strong base pairing stability, while those with low growth temperature have miRNAs with weaker base pairing.

Some miRNAs are conserved across species [33,34,35,36,37]. However, miRNAs with the same sequence conserved in different organisms with different body temperatures are unlikely to show the same silencing activities, as base pairing stability is strongly affected by temperature.

## 5. Perspectives

Biological functions are regulated by the environmental temperature, which influences the biochemical reactions inside the cell. A special characteristic of temperature is its pervasiveness; it can affect virtually all macromolecules in the cell. As behavioral, morphological, physiological, and other variable functions can be regulated by temperature, it was predicted that evolutionary adaptation of these phenomena would be observed.

As temperature is a key regulator of base pairing stability, it was also expected that the nucleotide composition in the genomes of organisms grown at higher temperatures would be selected for a higher proportion of G + C contents than A + T contents because the number of hydrogen bonds between G and C nucleotides (three hydrogen bonds) is higher compared to that of A and T nucleotides (two hydrogen bonds) and increases the thermodynamic stability. Unexpectedly, however, recent extensive sequencing of entire genomes revealed no obvious correlation between the genomic G + C content and the optimal growth temperature [38,39,40]. The thermostability of the double-stranded structure of DNA is conserved by reverse gyrase [41] or selection for certain dinucleotides [42] in thermophiles.

As there is no correlation between the G + C content of genomic DNA and growth temperature at least within prokaryotic genomes, it was considered that there would also be no such correlation at the RNA level. In bacteria, most genomic DNA is protein coding, so no such correlation has been found for protein-coding mRNAs either [38]. However, there is a correlation between the G + C content of structured RNAs (e.g., ribosomal and tRNAs) and growth temperature [38]. The G + C enrichment in structured RNAs represents striking evidence for selection in order to increase the thermostability of the corresponding regions by changing the nucleotide composition; as such, natural selection increasing the G + C content is observed in double-stranded regions of rRNA molecules, while the G + C content is decreased in single-stranded regions to maintain the single-stranded state in prokaryotes [39]. Thus, the correlation between G + C content and growth temperature is considered to be obvious for structured RNA molecules but not DNA duplexes. One possible reason is that a single nucleotide mutation would have a much greater effect on the thermodynamic stability of an RNA molecule than genomic DNA. Although the thermodynamic properties of 7-mer tRNA sequences in various species with different growth temperatures calculated by the nearest-neighbor procedure did not show an obvious correlation with growth temperature in our analysis, miRNA seed regions showed strong correlations with growth temperature [31], suggesting that evolutionary pressure is also added to miRNAs with secondary structures. However, RNA silencing efficiency is more strongly affected by the seed region than the 5′-terminal region, suggesting that evolutionary pressure on the miRNA 5′-terminal region is weak. Furthermore, our results cannot exclude other factor(s), such as the biogenesis procedure of siRNA/miRNA or the hydrolytic activity of nucleotide, as the other determinant(s) of the efficiency of RNA silencing.

## Abbreviations

siRNA	small interfering RNA
miRNA	microRNA
UTR	untranslated region
ncRNA	non-coding RNA
pri-miRNA	primary miRNA
pre-miRNA	precursor miRNA
AGO	Argonaute
RISC	RNA-induced silencing complex
CDS	coding sequence
RNAi	RNA interference
*T*m	melting temperature
